# Supplementary material for: A Metabolomics Investigation of the Metabolic Changes of Raji B Lymphoma Cells Undergoing Apoptosis Induced by Zinc Ions
Source: Metabolites. 2021 Oct 7;11(10):689. doi: 10.3390/metabo11100689 (PMC8540409; doi:10.3390/metabo11100689)
Supplement: Supplementary file 1 [file metabolites-11-00689-s001.zip › metabolites-1394431-supplementary.pdf]

A metabolomics investigation of the metabolic changes of Raji B lymphoma cells undergoing apoptosis induced by zinc ions

Naeun Yoon [1,2], Hyunbeom Lee [1], Geonhee Lee [3], Eun Hye Kim [4], Seong Hwan Kim [4], Jeong-O Lee [3], Yunseon Song [2], Jinyoung Park [1], So-Dam Kim [2], Yeojin Kim [2] and Byung Hwa Jung [1,5,*]

[1] Molecular Recognition Research Center, Korea Institute of Science and Technology, Seoul 02792, Korea

[2] College of Pharmacy, Sookmyung Women's University, Seoul 04310, Korea

[3] Advanced Materials Division, Korea Research Institute of Chemical Technology, Daejeon 34114, Korea

[4] Drug Discovery Platform Research Center, Department of Drug Discovery, Korea Research Institute of Chemical Technology, Daejeon 34114, Korea

[5] Division of Bio-Medical Science and Technology, KIST School, Korea University of Science and Technology (UST), Seoul 02792, Korea

Positive mode

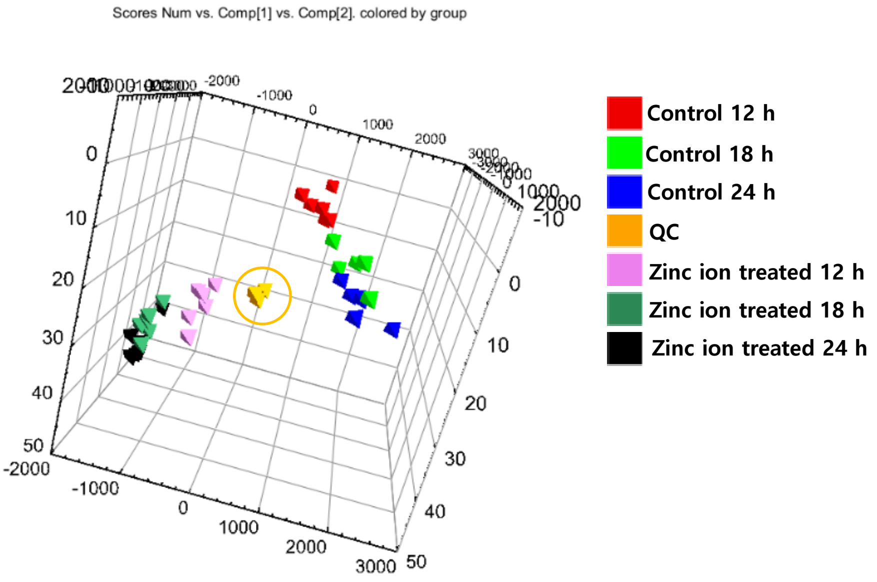

Negative mode

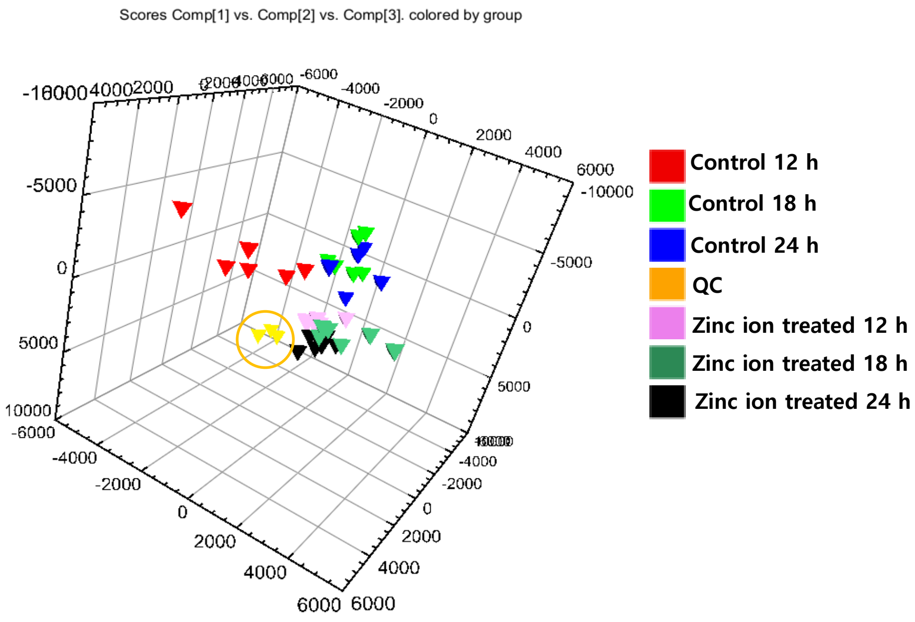

Supplement Figure S1. Principal Component Analysis (PCA) plot of quality control (QC) and samples. Positive mode (top) and negative mode (bottom).

Cleaved Caspase 3

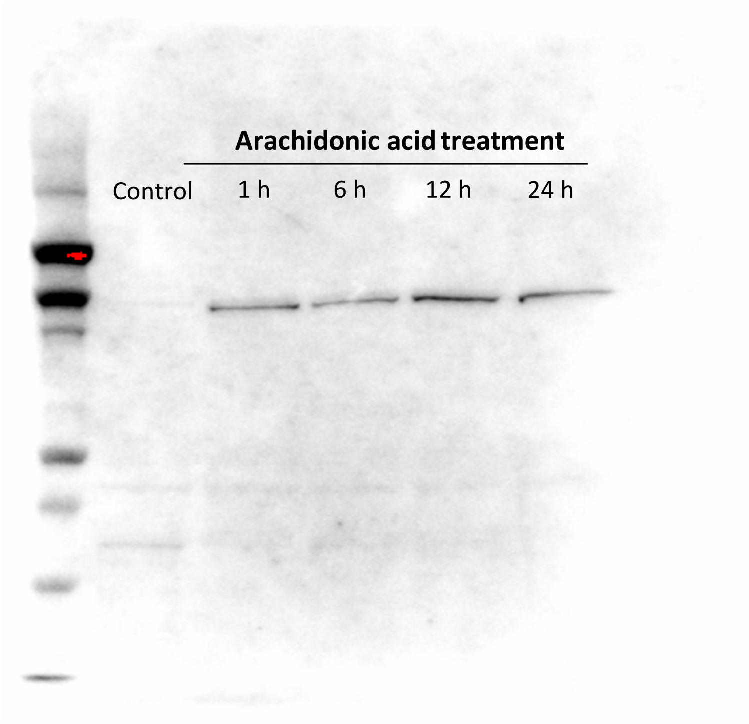

Cleaved PARP

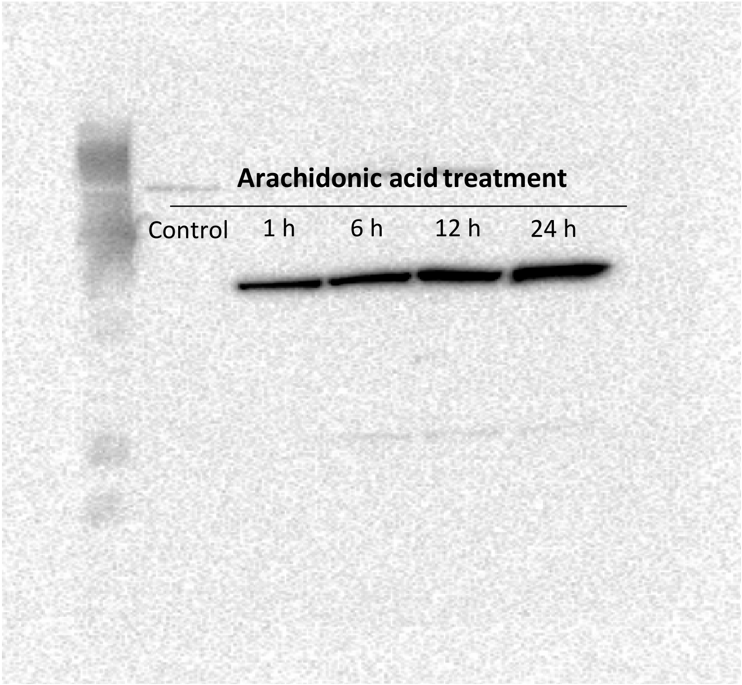

Supplement Figure S2. Uncropped original western blot images of figure 6C.

Cleaved Caspase 9

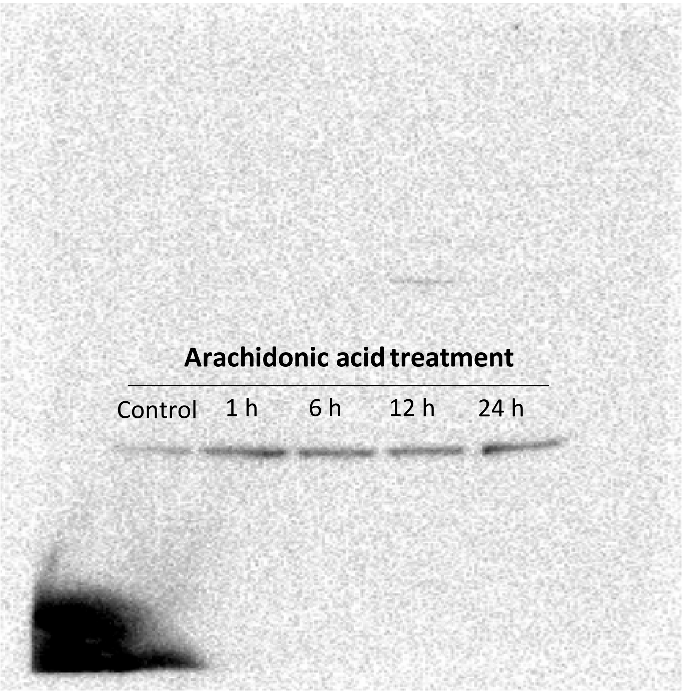

β-actin

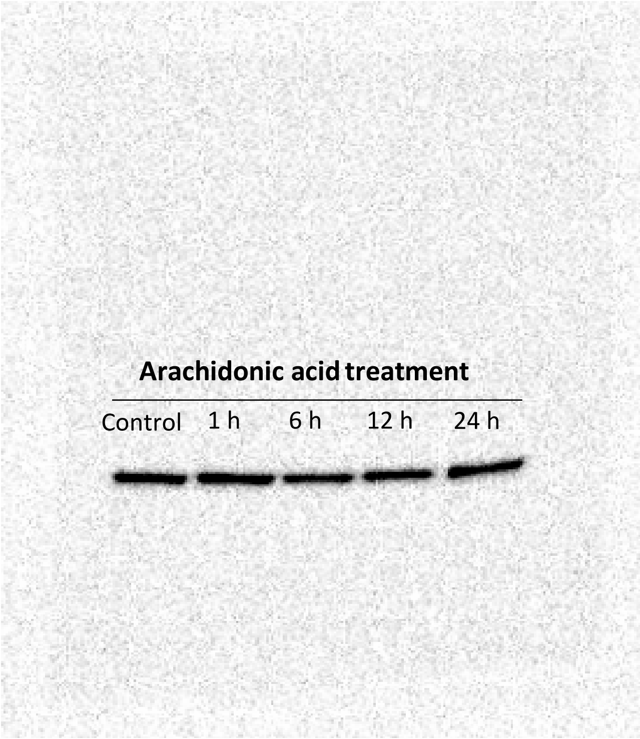

Supplement Figure S2. Continued.

Cleaved PARP

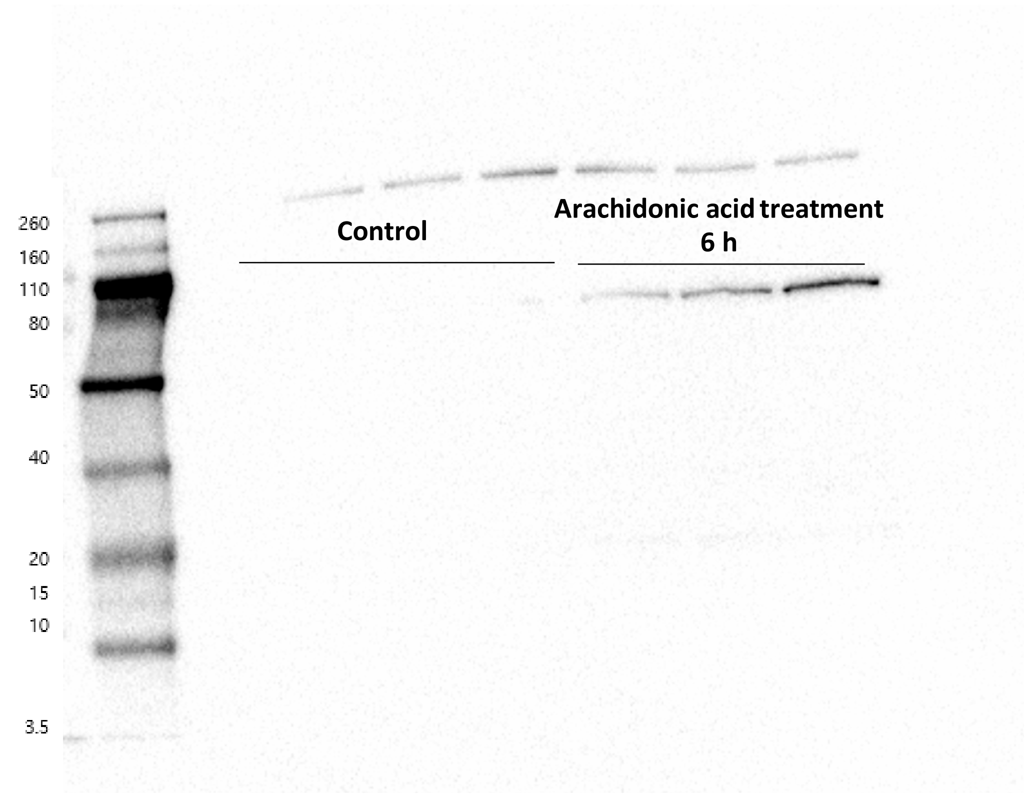

β-actin

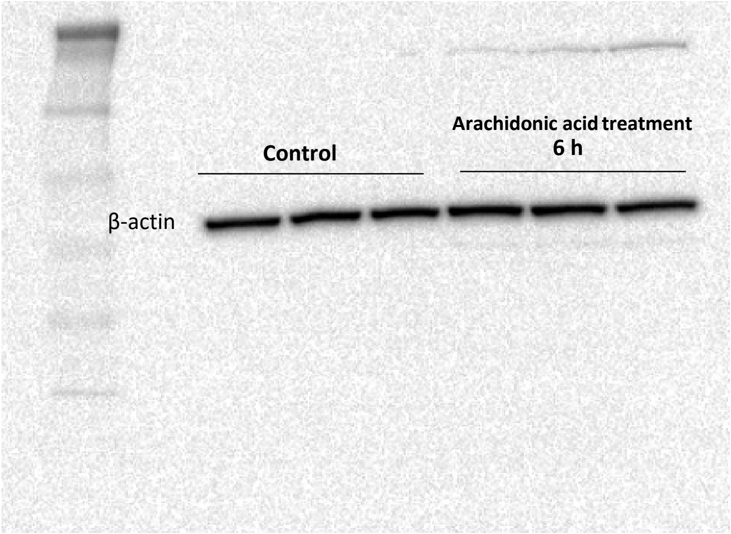

Supplement Figure S3. Uncropped original western blot images of figure 6D.

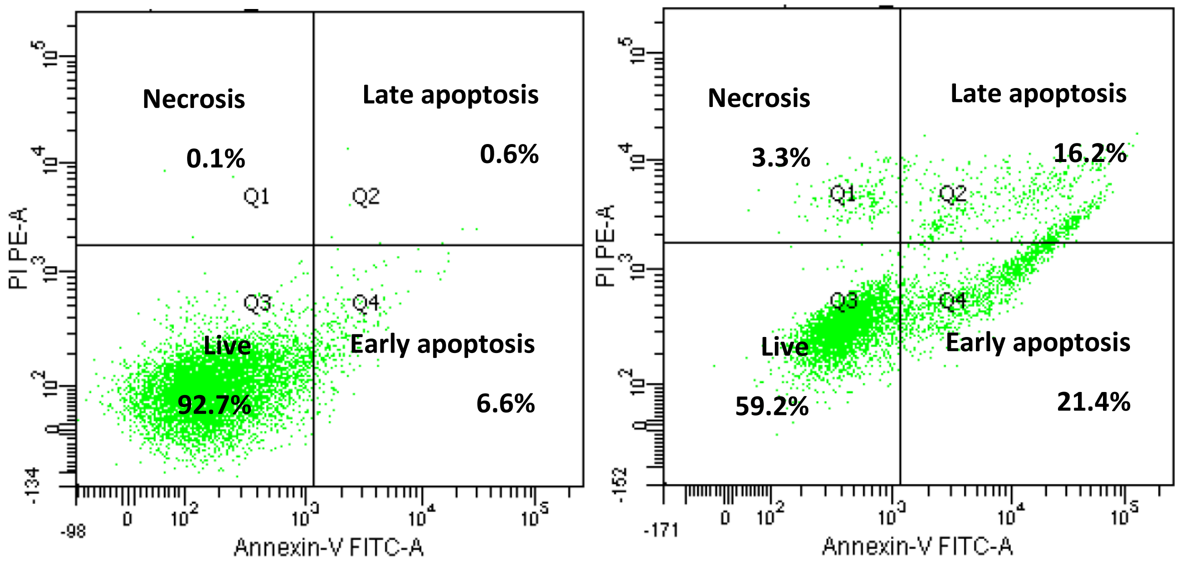

Supplement Figure S4. Apoptotic cell population evaluated by flow cytometry analysis following double staining with Annexin−V and propidium iodide (PI). Control group (left) and Raji cells treated with arachidonic acid 20 μM for 24 h (right).

Table S1. Significantly changed metabolites after Zn ion treatment. Student's t-test was assessed and statistically processed for each time point. Ratio means normalized area ratio of each metabolite between control and Zn ion treated group (normalized intensity of Zn ion treated group/normalized intensity of contraol group)

Class	Metabolite	zinc 12 h			zinc 18 h			zinc 24 h		
		P-value	Trend	Ratio	P-value	Trend	Ratio	P-value	Trend	Ratio
Amines	N,N'-Dicyclohexylurea	2.5E-04	↑	1.81	7.3E-04	↑	1.92	1.7E-01	↑	1.419
Amino acids, peptides, and analogues	Glutathione	2.1E-02	↑	1.51	1.4E-04	↓	0.304	1.6E-02	↓	0.204
Amino acids, peptides, and analogues	Proline	8.8E-02	↑	1.48	2.9E-03	↓	0.116	2.7E-03	↓	0.114
Amino acids, peptides, and analogues	Leucine	8.4E-01	↑	1.04	6.7E-03	↓	0.0868	4.9E-04	↓	0.0551
Amino acids, peptides, and analogues	Threonic acid	6.1E-03	↑	2.16	8.6E-03	↑	1.63	2.0E-01	↑	1.62
Amino acids, peptides, and analogues	N-Acetyl-L-alanine	9.0E-03	↑	3.04	6.4E-03	↓	0.550	7.6E-08	↓	0.147
Amino acids, peptides, and analogues	L-Methionine	3.3E-04	↑	4.61	7.4E-03	↓	0.290	3.7E-04	↓	0.0855
Amino acids, peptides, and analogues	L-Histidine	1.1E-05	↑	2.58	2.1E-03	↓	0.181	4.3E-07	↓	0.0542
Amino acids, peptides, and analogues	L-Carnitine	9.1E-02	↑	1.13	4.6E-03	↓	0.529	1.9E-02	↓	0.167
Amino acids, peptides, and analogues	L-Phenylalanine	1.6E-03	↑	2.30	1.6E-02	↓	0.328	1.9E-07	↓	0.169
Amino acids, peptides, and analogues	L-Arginine	2.9E-03	↑	3.63	6.7E-04	↓	0.531	7.6E-03	↓	0.223
Amino acids, peptides, and analogues	N-Acetyl-DL-methionine	1.6E-05	↓	0.182	2.0E-04	↓	0.0316	1.2E-09	↓	0.0801
Amino acids, peptides, and analogues	L-Tryptophan	1.5E-04	↑	2.90	3.3E-05	↓	0.423	2.1E-06	↓	0.114
Amino acids, peptides, and analogues	Pantothenic acid	1.7E-05	↑	3.36	1.4E-06	↓	0.0676	6.5E-08	↓	0.137
Amino acids, peptides, and analogues	Phe-Tyr	2.6E-03	↑	1.87	6.0E-01	↓	0.860	2.4E-04	↓	0.415
Benzene and substituted derivatives	N-formylanthranilic acid	1.2E-03	↑	3.07	5.8E-03	↑	1.02	3.6E-03	↓	0.375
Benzene and substituted derivatives	Phenyllactic acid	6.5E-06	↓	0.0410	2.3E-04	↓	0.0410	5.0E-06	↓	0.00396
Benzene and substituted derivatives	Hippuric acid	1.0E-07	↓	0.204	4.1E-04	↓	0.0209	5.5E-10	↓	0.146
Benzene and substituted derivatives	DL-Indole-3-lactic acid	2.9E-04	↓	0.0231	4.4E-05	↓	3.52E-03	1.7E-06	↓	1.45E-03
Carboxylic acids and derivatives	Sulfinoalanine	7.9E-04	↑	5.90	1.1E-05	↓	0.0136	2.0E-08	↓	0.0132
Hydroxy acids and derivatives	Malic acid/fumaric acid	1.4E-03	↓	0.306	1.8E-01	↓	0.755	4.4E-03	↓	0.111
Organonitrogen compounds	Spermidine	9.3E-02	↓	0.788	6.6E-06	↓	0.259	3.0E-08	↓	0.132

Organooxygen compounds	Arabinose 5-phosphate	6.7E-03	↑	4.59	1.2E-02	↓	0.431	3.5E-03	↓	0.0671
Organonitrogen compounds	Phosphorylcholine	4.9E-02	↑	1.90	4.9E-01	↓	0.818	4.9E-02	↓	0.584
Organosulfonic acids and derivatives	Taurine	1.3E-01	↑	1.57	4.8E-02	↓	0.672	2.5E-01	↓	0.461
Fatty Acyls	Carnosine (beta-alanyl-L-histidine)	9.3E-02	↑	2.20	9.1E-06	↓	0.262	4.7E-08	↓	0.117
Fatty Acyls	Acetylcarnitine	2.4E-03	↑	3.82	3.2E-03	↓	0.857	3.4E-01	↓	0.823
Fatty Acyls	3-Carboxy-4-methyl-5-propyl-2-	2.0E-04	↑	2.33	4.8E-02	↑	3.62	8.5E-01	↑	1.04
Fatty Acyls	Butyryl-L-carnitine	7.4E-03	↑	2.41	2.9E-04	↓	0.0178	1.7E-07	↓	0.0937
Fatty Acyls	Palmitic acid	1.6E-07	↑	2.28	2.0E-03	↑	1.41	7.7E-03	↓	0.672
Fatty Acyls	Isovalerylcarnitine	1.0E-01	↓	0.637	2.7E-05	↓	0.223	1.3E-01	↓	0.462
Fatty Acyls	14-fluoro-myristic acid	9.1E-06	↑	1.98	5.9E-05	↑	1.96	1.3E-01	↑	1.50
Fatty Acyls	Oleic acid	1.6E-03	↓	0.069	4.8E-02	↓	0.0164	2.9E-03	↓	0.00487
Fatty Acyls	Vaccenic acid	5.1E-04	↑	1.86	5.6E-01	↑	1.22	5.2E-05	↓	0.257
Fatty Acyls	Stearic acid	3.1E-06	↑	1.97	1.5E-02	↑	1.61	5.2E-02	↓	0.730
Fatty Acyls	Hexanoylcarnitine	1.4E-06	↑	4.71	2.4E-03	↓	0.158	2.9E-03	↓	0.0256
Fatty Acyls	Arachidonic acid	2.9E-03	↑	84.7	5.1E-03	↑	42.3	2.6E-03	↑	16.5
Fatty Acyls	(±)12,13-DiHOME	2.9E-04	↑	1.97	1.8E-02	↑	1.91	3.7E-01	↑	1.09
Fatty Acyls	(4Z,7Z,10Z,13Z,16Z,19Z)-4,7,10,13,1	1.7E-03	↑	43.3	4.8E-03	↑	7.65	3.1E-03	↑	3.88
Fatty Acyls	7Z, 10Z, 13Z, 16Z, 19Z-	1.5E-02	↑	46.1	2.0E-02	↑	6.47	1.7E-02	↑	3.37
Fatty Acyls	Adrenic acid	4.6E-03	↑	37.5	4.1E-03	↑	5.10	5.8E-03	↑	3.61
Fatty Acyls	Palmitoyl-L-carnitine	3.6E-02	↓	0.0670	2.3E-05	↓	0.0322	2.0E-04	↓	0.0262
Glutamic acid and derivatives	Folic acid	1.6E-01	↓	0.859	5.8E-04	↑	1.65	7.1E-02	↓	0.868
Glutamic acid and derivatives	Glutamate	5.8E-05	↑	2.91	4.4E-03	↓	0.960	6.6E-02	↓	0.680
Glycerophospholipids	1-Stearoylglycerophosphocholine	1.4E-04	↑	4.67	2.0E-04	↑	1.80	1.4E-01	↑	1.58
Glycerophospholipids	Glycerophosphocholine	7.3E-04	↑	1.13E+03	7.0E-06	↑	1.04E+03	1.2E-01	↑	5.75E+01
Glycerophospholipids	Lyso phosphatidylcholine 16:1	4.2E-03	↑	2.01	3.5E-05	↑	1.57	6.6E-01	↑	1.11
Glycerophospholipids	Phosphatidylcholine (0:0/18:0)	3.8E-04	↑	4.17	6.2E-03	↑	1.87	1.9E-01	↑	1.18
Glycerophospholipids	Phosphatidylcholine 14:0-18:2	5.7E-04	↑	1.60	8.7E-03	↑	1.76	1.8E-01	↓	0.846
Glycerophospholipids	Phosphatidylcholine 16:0-16:1	4.4E-03	↑	1.27	1.6E-01	↑	1.28	5.2E-02	↓	0.737
Glycerophospholipids	Phosphatidylcholine 36:4	1.7E-04	↑	10.1	8.0E-06	↑	6.82	3.0E-02	↑	9.19

Glycerophospholipids	Phosphatidylcholine lyso 16:0	5.9E-05	↑	2.69	1.4E-01	↓	0.894	1.2E-02	↓	0.724
Glycerophospholipids	Phosphatidylethanolamine	3.1E-04	↑	1.84	1.5E-01	↓	0.7549	3.7E-02	↓	0.235
Glycerophospholipids	Phosphatidylethanolamine lyso 18:0	3.7E-03	↑	1.86	4.3E-01	↓	0.894	6.4E-03	↓	0.468
Glycerophospholipids	Phosphatidylethanolamine lyso alkenyl	2.4E-04	↓	0.646	3.6E-02	↓	0.262	1.3E-03	↓	0.127
Glycerophospholipids	Phosphatidylinositol lyso 18:0	3.2E-03	↑	3.40	1.3E-01	↑	1.73	8.9E-05	↓	0.410
Glycerophospholipids	sn-Glycerol 3-phosphate	9.8E-07	↑	2.66	1.4E-02	↑	1.59	9.2E-05	↓	0.586
Nucleosides, nucleotides, and analogues	5′-Deoxy-5′-(methylthio)adenosine	7.0E-06	↑	1.73	1.0E-07	↑	1.37	2.1E-03	↓	0.398
Nucleosides, nucleotides, and analogues	Adenosine monophosphate	2.7E-05	↑	36.4	9.8E-07	↓	0.100	2.7E-06	↓	0.116
Nucleosides, nucleotides, and analogues	ADP	6.7E-04	↓	0.390	2.7E-03	↓	0.434	7.7E-07	↓	0.384
Nucleosides, nucleotides, and analogues	Fructose/Lactic acid(M-H)	2.0E-01	↑	1.39	2.2E-04	↓	0.395	2.9E-06	↓	0.246
Nucleosides, nucleotides, and analogues	GMP	5.8E-03	↓	0.243	1.3E-04	↑	2.52	8.8E-08	↓	0.240
Nucleosides, nucleotides, and analogues	Inosine	2.1E-03	↑	8.59	3.4E-02	↑	1.57	3.4E-06	↓	0.221
Nucleosides, nucleotides, and analogues	Inosine-5'-monophosphate	5.0E-05	↑	15.9	6.6E-04	↑	2.84	2.0E-06	↓	0.212
Nucleosides, nucleotides, and analogues	N6-(1,2-dicarboxyethyl)-AMP	1.5E-04	↓	0.0228	5.6E-02	↓	0.291	3.6E-04	↓	0.0326
Nucleosides, nucleotides, and analogues	Succinic acid	5.2E-04	↓	0.0577	1.4E-02	↓	0.0462	1.9E-03	↓	0.0721
Nucleosides, nucleotides, and analogues	Uridine	1.1E-04	↓	0.0577	1.1E-03	↓	0.234	2.2E-05	↓	0.0388
Nucleosides, nucleotides, and analogues	Uridine monophosphate (UMP)	1.1E-04	↓	0.0397	9.4E-05	↑	2.14	8.9E-04	↓	0.143

Table S2. The information of identified metabolites in metabolomics study. The Metabolomics Standards Initiative (MSI), retention time (RT), ionization mode, adduct, and used database are shown in the below.

Class	Identified metabolites	RT	Adduct	M/Z	Mode	MSI chemical identification level	Database
Amines	N,N'-Dicyclohexylurea	10.82	M+H	225.19	positive	level 3	METLIN
Amino acids, peptides, and analogues	Glutathione	1.45	M+H	308.08	positive	level 2	HMDB
Amino acids, peptides, and analogues	L-Arginine	0.99	M+H	175.11	positive	level 2	HMDB
Amino acids, peptides, and analogues	L-Carnitine	1.03	M+H	162.10	positive	level 2	HMDB
Amino acids, peptides, and analogues	Leucine	1.05	M+H	132.06	positive	level 2	HMDB
Amino acids, peptides, and analogues	L-Histidine	0.99	M-H	154.06	negative	level 2	METLIN
Amino acids, peptides, and analogues	L-Methionine	1.40	M+H	150.05	positive	level 2	MASSBANK
Amino acids, peptides, and analogues	L-Phenylalanine	4.77	M+H	166.08	positive	level 2	METLIN
Amino acids, peptides, and analogues	L-Tryptophan	6.24	M+H	188.06	positive	level 2	MASSBANK
Amino acids, peptides, and analogues	N-Acetyl-DL-methionine	6.44	M-H	190.05	negative	level 2	METLIN
Amino acids, peptides, and analogues	N-Acetyl-L-alanine	2.22	M-H	130.09	negative	level 2	METLIN
Amino acids, peptides, and analogues	Pantothenic acid	5.89	M+H	220.11	positive	level 2	HMDB
Amino acids, peptides, and analogues	Phe-Tyr	14.73	M-H	327.13	negative	level 2	METLIN
Amino acids, peptides, and analogues	Proline	1.04	M+H	116.06	positive	level 2	HMDB
Amino acids, peptides, and analogues	Threonic acid	1.71	M+H	137.04	positive	level 2	HMDB
Benzene and substituted derivatives	DL-Indole-3-lactic acid	8.08	M+H	206.07	positive	level 2	MASSBANK
Benzene and substituted derivatives	Hippuric acid	7.37	M-H	178.05	negative	level 2	METLIN
Benzene and substituted derivatives	N-formylanthranilic acid	1.32	M+H	166.04	positive	level 3	METLIN
Benzene and substituted derivatives	Phenyllactic acid	8.00	M-H	165.06	negative	level 2	METLIN
Carboxylic acids and derivatives	Sulfinoalanine	0.99	M+H	154.02	positive	level 2	METLIN

Fatty Acyls	(±)12,13-DiHOME	12.69	M-H	313.24	negative	level 2	MASSBANK
Fatty Acyls	(4Z,7Z,10Z,13Z,16Z,19Z)-4,7,10,13,1 6,19-Docosahexaenoic acid	15.19	M-H	327.23	negative	level 1	MASSBANK
Fatty Acyls	14-fluoro-myristic acid	10.82	M+H	247.17	positive	level 3	METLIN
Fatty Acyls	3-Carboxy-4-methyl-5-propyl-2-furanpropanoic acid	12.15	M-H	239.07	negative	level 2	MASSBANK
Fatty Acyls	7Z, 10Z, 13Z, 16Z, 19Z-docosapentaenoic acid	15.43	M-H	329.25	negative	level 2	METLIN
Fatty Acyls	Acetylcarnitine	1.05	M+H	204.12	positive	level 2	HMDB
Fatty Acyls	Adrenic acid	15.77	M-H	331.26	negative	level 2	METLIN
Fatty Acyls	Arachidonic acid	15.22	M-H	303.23	negative	level 1	In house database
Fatty Acyls	Butyryl-L-carnitine	5.48	M+H	232.15	positive	level 2	METLIN
Fatty Acyls	Carnosine (beta-alanyl-L-histidine)	0.86	M+X	156.07	positive	level 2	MASSBANK
Fatty Acyls	Hexanoylcarnitine	7.63	M+H	260.18	positive	level 2	HMDB
Fatty Acyls	Isovalerylcarnitine	6.57	M+H	246.16	positive	level 2	HMDB
Fatty Acyls	Oleic acid	13.20	M-H	281.25	negative	level 2	MASSBANK
Fatty Acyls	Palmitic acid	15.56	M-H	255.23	negative	level 2	MASSBANK
Fatty Acyls	Palmitoyl-L-carnitine	13.00	M+H	400.33	positive	level 2	METLIN
Fatty Acyls	Stearic acid	16.20	M-H	283.26	negative	level 2	HMDB
Fatty Acyls	Vaccenic acid	15.73	M-H	281.25	negative	level 2	MASSBANK
Glutamic acid and derivatives	Folic acid	7.06	M-H	440.13	negative	level 2	MASSBANK
Glutamic acid and derivatives	Glutamate	1.20	M+H	148.04	positive	level 2	HMDB
Glycerophospholipids	1-Stearoylglycerophosphocholine	15.18	M+H	524.35	positive	level 2	MASSBANK
Glycerophospholipids	Glycerophosphocholine	1.14	M+H	258.10	positive	level 2	METLIN
Glycerophospholipids	phosphatidylcholine lyso 16:1	11.48	M+H	494.15	positive	level 2	MASSBANK
Glycerophospholipids	Phosphatidylcholine (0:0/18:0)	15.19	M+FA-H	568.36	negative	level 2	MASSBANK
Glycerophospholipids	Phosphatidylcholine 14:0-18:2	17.18	M+FA-H	774.52	negative	level 2	MASSBANK
Glycerophospholipids	Phosphatidylcholine 16:0-16:1	14.44	M+FA-H	452.28	negative	level 2	MASSBANK

Glycerophospholipids	Phosphatidylcholine 36:4	17.77	M+H	782.54	positive	level 3	MASSBANK
Glycerophospholipids	Phosphatidylcholine lyso 16:0	14.41	M+FA-H	540.33	negative	level 2	MASSBANK
Glycerophospholipids	Phosphatidylethanolamine (18:1(9Z)/0:0)	14.64	M+H	480.29	positive	level 2	METLIN
Glycerophospholipids	Phosphatidylethanolamine lyso 18:0	15.21	M-H	480.31	negative	level 2	MASSBANK
Glycerophospholipids	Phosphatidylethanolamine lyso alkenyl 16:0	14.81	M-H	436.28	negative	level 2	MASSBANK
Glycerophospholipids	Phosphatidylinositol lyso 18:0	16.83	M-H	599.31	negative	level 2	MASSBANK
Glycerophospholipids	sn-Glycerol 3-phosphate	1.14	M-H	171.01	negative	level 2	MASSBANK
Hydroxy acids and derivatives	Malic acid/fumaric acid (21, neg)	1.92	M-H2O-H	115.00	negative	level 2	METLIN
Nucleosides, nucleotides, and analogues	5′-Deoxy-5′-(methylthio)adenosine	6.56	M+H	298.09	positive	level 2	HMDB
Nucleosides, nucleotides, and analogues	Adenosine monophosphate	1.55	M+H	348.06	positive	level 2	HMDB
Nucleosides, nucleotides, and analogues	ADP (245, neg)	1.41	M-H	426.02	negative	level 2	MASSBANK
Nucleosides, nucleotides, and analogues	Fructose/Lactic acid(M-H)	1.59	M-2H	89.02	negative	level 3	METLIN
Nucleosides, nucleotides, and analogues	GMP	1.71	M-H	362.05	negative	level 2	METLIN
Nucleosides, nucleotides, and analogues	Inosine	4.37	M-H	267.07	negative	level 2	METLIN
Nucleosides, nucleotides, and analogues	Inosine-5'-monophosphate	1.72	M-H	347.04	negative	level 2	METLIN
Nucleosides, nucleotides, and analogues	N6-(1,2-dicarboxyethyl)-AMP	5.33	M-H	462.07	negative	level 2	METLIN
Nucleosides, nucleotides, and analogues	Succinic acid	2.21	M-H	117.02	negative	level 2	METLIN
Nucleosides, nucleotides, and analogues	Uridine	2.29	M-X	306.06	negative	level 2	MASSBANK
Nucleosides, nucleotides, and analogues	Uridine monophosphate (UMP)	1.42	M-H	323.03	negative	level 2	METLIN
Organonitrogen compounds	Phosphorylcholine	1.06	M+H	184.07	positive	level 2	METLIN
Organonitrogen compounds	Spermidine	1.03	M+H	146.11	positive	level 2	MASSBANK
Organooxygen compounds	Arabinose 5-phosphate	1.23	M-H2O-H	211.00	negative	level 2	MASSBANK/METLIN
Organosulfonic acids and derivatives	Taurine	1.11	M+H	126.02	positive	level 2	METLIN
